# Retrospective Cross‐Sectional Study of Occupational Infection Risk With Zoonotic Pathogens in Austrian Veterinary Practitioners in the Year 2022

**DOI:** 10.1002/vms3.70485

**Published:** 2025-08-06

**Authors:** Tatjana Sattler, Bernhard Benka, Irene Zimpernik, Sarah Pürrer, Karoline Waldner, Georg G. Duscher, Agnes Kuffer‐Rosewick, Jasmin Fertey, Sebastian Ulbert, Friedrich Schmoll

**Affiliations:** ^1^ Austrian Agency for Health and Food Safety (AGES) Mödling Austria; ^2^ Clinic for Ruminants and Swine University Leipzig Leipzig Germany; ^3^ Austrian Agency for Health and Food Safety (AGES) Vienna Austria; ^4^ Austrian Agency for Health and Food Safety (AGES) Innsbruck Austria; ^5^ Fraunhofer Institute for Cell therapy and Immunology Leipzig Germany

**Keywords:** Austria, flavivirus, hepatitis E, MRSA, veterinarians

## Abstract

**Objective:**

The aim of the study was to determine the prevalence of antibodies against hepatitis E virus (HEV), tick‐borne encephalitis virus (TBEV), West Nile virus (WNV), Crimean–Congo haemorrhagic fever (CCHF) virus, rabies virus, *Echinococcus* spp.*, Brucella* spp., as well as the colonisation with methicillin‐resistant *Staphylococcus aureus* (MRSA) among Austrian veterinarians in dependence on their field of occupation, age, gender and, if applicable, the vaccination status.

**Methods and results:**

A total of 293 Austrian veterinary practitioners were included in the study. All study participants had to fill in a questionnaire regarding personal details, field of occupation and vaccination status against TBEV and rabies. Furthermore, a nasal swab for detection of colonisation with MRSA and a blood sample were taken. Antibodies against HEV, TBEV, WNV, CCHF virus and *Echinococcus* spp. were analysed by ELISA tests. Antibodies against *Brucella* spp. were measured by complement fixation test. Rabies antibodies were analysed by virus neutralisation test. Most study participants were vaccinated against rabies (93.2%) and TBEV (94.3%). Accordingly, a high prevalence of TBEV antibodies was found (95.9%). HEV antibodies were detected in 11.3% of the study participants, with a significantly higher seroprevalence in the oldest age group (23.3%). MRSA colonisation was age‐depended and significantly higher in study participants with occupational livestock exposure (10.3% vs. 2.2%). Hygienic measures such as hand washing and disinfection led to less MRSA colonisation. Antibodies against WNV were found in 3.1% of the study participants. No antibodies against *Brucella* spp., *Echinococcus* spp. and CCHF were detected.

**Conclusions:**

Despite the limitations of the study design, the included veterinarians represent the Austrian average veterinarians regarding gender, age and field of occupation. The vaccination rate against TBEV and rabies was high. HEV seroprevalence was lower than in other studies, whereas MRSA colonisation rates and WNV seroprevalence were comparable to results of other European studies.

## Graphical abstract

• Vaccination rates against rabies and TBEV and accordingly the respective seroprevalence rates exceed 90% among Austrian veterinary practitioners.

• The prevalence of antibodies against HEV and the colonisation with MRSA increases with the age of the study participants from 6.2%/6.2% in the youngest age group to 23.3%/21.9% in the oldest group.

• MRSA colonisation in Austrian veterinarians is significantly higher in those with livestock occupational exposure (10.3% vs. 2.2%) and is lower among those that wash and/or disinfect their hands (5.2% vs. 66.3%).

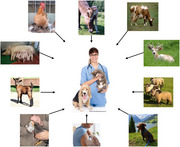

## 1 Introduction

In the course of their professional occupation, veterinarians are exposed to a multitude of pathogens. Relevant for human health in this context are pathogens with zoonotic potential, namely pathogens that can be transmitted from animal to human and vice versa. The relevance of the respective zoonotic pathogens depends on the field of veterinary activity. Zoonotic agents that can concern veterinarians are, among others, hepatitis E virus (HEV), *Brucella* sp., methicillin‐resistant *Staphylococcus aureus* (MRSA), West Nile virus (WNV), Tick‐borne Encephalitis virus (TBEV), Crimean–Congo haemorrhagic fever (CCHF) virus, *Echinococcus* spp. and rabies virus (Baker and Grey 2009; Jackson and Villarroel 2012).

Infections with HEV in western countries are mainly subclinical and caused by the genotype 3 of the virus. Sporadic clinical apparent hepatitis, however, can occur particularly in persons with pre‐existing liver damage or immune suppression. According to the European Food Safety Authority (EFSA), the intake of raw or undercooked pork is the main risk for infection with HEV. Furthermore, those working in direct contact with infected animals are at an elevated risk (EFSA Panel on Biological Hazards et al., Ricci et al. 2017).

*Brucella* spp. are reckoned to be pathogens with a high zoonotic potential. Relevant are *Brucella abortus* and *Brucella melitensis* as cause of brucellosis in cattle, sheep and goat herds, but also *Brucella suis* and *Brucella canis* with pigs or dogs, respectively, as main hosts. Although the zoonotic potential of *Brucella suis* is considered to be low, all mentioned *Brucella* spp. can cause severe disease in human (Pinn‐Woodcock et al. 2023).

Livestock‐associated (LA‐) MRSA are highly prevalent in pigs and cattle, but also in sheep, goats, poultry and buffalos worldwide (Abdullahi et al. 2023). Consequently, workers in close contact with livestock, especially farmers and veterinarians, are at greater risk for colonisation with LA‐MRSA leading to possible subsequent infections (Springer et al. 2009; Chen and Wu 2020).

WNV and TBEV are flaviviruses mainly transmitted by mosquitoes and ticks, respectively, and occur worldwide. Antibodies against TBEV can be found in livestock and wild mammals, in some cases clinical symptoms in animals have been described (Michelitsch et al. 2019). TBEV infections after the consumption of raw milk or milk products are also known (Ličková et al. 2021). The risk of the occurrence of WNV in central Europe increases due to climate change, and yearly infections are documented from a variety of European countries, including Austria and Germany. While most human infections are clinically inapparent or mild, some can lead to severe neurological symptoms and death (Erazo et al. 2024).

CCHF is a tick‐borne disease. The virus, however, can also be transmitted via contact with infected slaughter animals or nosocomially due to contact with an ill person. Until now, the northern borderline is the 50th latitude, but the distribution of the transmitting *Hyalomma* tick is expanding northwards. The CCHF virus can cause a severe disease with a mortality of around 30% (Wiemer 2015). An occupational risk for infection regarding veterinarians and workers with close animal contact in endemic regions has been discussed (Aydin et al. 2020).

*Echinococcus multilocularis* is endemic in Europe. Several studies on foxes revealed an overall prevalence of 8.35% for Austria with a higher prevalence of up to 50% observed in the western part of the country (Duscher et al. 2006). In Austria, about 10–24 human cases of alveolar echinococcosis are reported annually (AGES 2023). *Echinococcus granulosus* in humans in Austria is reported in approximately the same range, e.g., 30 cases in 2022. The latter can mostly be linked to travel history and therefore is not supposed to be endemic in Austria (AGES 2023).

Rabies is a fatal zoonotic disease that can be transmitted from affected mammals to humans. Although an effective post‐exposure prophylaxis is available, a pre‐exposure prophylaxis is recommended for persons with occupational risk (World Health Organization 2023). This recommendation has become more relevant since a case of failure of post‐exposure prophylaxis in an individual with immune suppression has been described recently (Holzbauer et al. 2023). In Austria, the government recommends the vaccination against rabies for veterinarians. Although most European countries have been declared free of urban rabies, the disease can re‐emerge as happened in Poland in 2021 (Smreczak et al. 2022).

Employers, including veterinary practitioners who employ assistants are, according to the law of the respective country, obligated to implement appropriate measures for risk prevention. According to a Finnish study, only about 8% of the participating veterinarians stated a good knowledge of zoonoses and their prevention, whereas about 91% had already been exposed to zoonotic pathogens (Kinnunen et al. 2022).

The aim of the study was to determine the prevalence of antibodies against HEV, TBEV, WNV, CCHF virus, rabies virus, *Echinococcus* spp. and *Brucella* spp., as well as the colonisation with MRSA among Austrian veterinarians in dependence on their field of occupation, age, gender and, if applicable, the vaccination status.

## 2 Materials and Methods

### 2.1 Study Population and Study Design

A total of 293 probands from all over Austria were included in the study. A 100 and one of them were recruited at a major Austrian veterinary science conference in September 2022. The remaining 192 were veterinarians that had been informed of the planned study via the veterinary press and were sampled in October and November 2022. Inclusion criteria were that the probands were veterinary practitioners with direct animal contact and consent to participate and to provide a professionally taken blood sample and nasal swab. All blood samples were taken by peripheral venipuncture according to standard operation procedure (Meyer et al. 2017). Furthermore, a questionnaire had to be filled in in its entirety and in an accurate and precise manner.

### 2.2 Questionnaire

All study participants had to complete a provided questionnaire. Queried were age, gender, nationality, duration of practical veterinary activity, time volume of work and kind of animal contact they have had during the course of their professional and private lives. Animal contact was divided in the following categories: small animals (cats, dogs), rodents, reptiles, birds, poultry, livestock animals (cattle, sheep and pigs), horses, aquatic animals and wildlife animals including hunting activities. Furthermore, information about travel history, hygienic habits during veterinary activity (washing and disinfection of hands, wearing gloves) and vaccination history against TBEV and rabies were queried. All data were analysed anonymously.

### 2.3 Laboratory Analyses

All serum samples were examined using commercially available ELISAs for detection of antibodies against HEV (HEV‐IgG Elisa, Fortress Diagnostics Limited, Antrim, UK), CCHF virus (ID Screen CCHF Double antigen Multi‐species Elisa, IDvet, Grabels, France), and *Echinococcus* spp. (*Echinococcosis multilocularis* IgG ELISA, Bordier Affinity Products SA, Crissier, Switzerland). All tests were carried out according to the manufacturer's instructions and the validity of the tests and the calculation of the results were determined in accordance with the instructions for use. In case of positive results in the *Echinococcus* ELISA, a Western Blot detecting antibodies against *Echinococcus multilocularis* and *Echinococcus granulosus* with a specificity of 95% was performed. All sera were tested for antibodies against WNV and TBEV using two ELISA test systems developed by the Fraunhofer Institute for Cell Therapy and Immunology (Leipzig, Germany) described in Rockstroh et al. (2019). In cases of positive or questionable results regarding specific WNV antibodies, a competition ELISA as described in Berneck et al. (2022) was conducted to confirm the results. Antibodies against *Brucella* spp. were measured using the complement fixation test (CFT), performed according to the standard operating procedure of the reference laboratory of the EU (ANSES 2021). If a serum showed an anti‐complementary reaction in the CFT, the Rose Bengal test was performed according to ANSES (2021). The determination of rabies specific antibody titres was conducted by a fluorescent antibody virus neutralisation test. The test, calculation and interpretation of the results were performed according to the World Organization for Animal Health (WOAH) manual (WOAH 2023).

The occurrence of MRSA was detected by swabbing the participant's anterior nose. For semi‐selective enrichment, the swabs were incubated in Müller–Hinton Broth 6.5% NaCl overnight at 36 ± 1°C in ambient air. Aliquots of 10 µL were streaked on MRSA2 Brilliance Agar (Oxoid) and incubated overnight at 37 ± 1°C. Presumptive MRSA were sub‐cultured on Columbia Sheep Blood Agar and cefoxitin susceptibility was tested. MRSA were confirmed by time‐of‐flight mass spectrometry (MALDI‐TOF MS) and PCR as described in Firth et al. (2022).

### 2.4 Statistical Analysis

Data were analysed descriptively regarding age, gender, duration of veterinary activity, kind of animal contact, vaccination against TBEV and rabies. The age of the participants was tested for normal distribution with the Kolmogorov–Smirnov test, mean and standard deviation (s.d.) were calculated. Age groups were formed according to mean age of the study. The age group of the older half of the participants was then further divided into two groups to get explicit information about the study population of 60 years or older. Where applicable, percentages and the 95% confidence interval (CI) using the method of Clopper and Pearson were calculated. The Fisher's exact test was used for determination of correlations between the parameters, i.e., laboratory results and potential risk factors, age groups, laboratory results and vaccination history. For differences of the rabies antibody titre between the groups, the Kruskal–Wallis test (in case of more than two groups), followed by the Mann–Whitney test were used. Results with *p* < 0.05 were considered statistically significant. All data were comprised in Microsoft Excel Version 2021. Data analyses were performed using IBM SPSS statistics (version 29).

## 3 Results

### 3.1 Study Population

Of the 293 study participants, 216 (73.7%, CI: 68.7%–78.7%) were female, 77 (26.3%, CI: 21.3%–31.3%) male. The age ranged from 21 to 83 years with a mean of 44.0 years (s.d. 11.3 years). Hence, the age groups were defined as under 44 years (*n* = 145), 44–60 (*n* = 129) and over 60 years (*n* = 19). Of the youngest age group, 84.1% (CI: 78.3%–90.1%) were female, whereas in the oldest age group, 84.2% (CI: 67.8%–100%) were male.

Whilst looking on the animal contact (Table 1), most veterinarians were regularly exposed to more than one species or animal group. Although 40.6% (CI: 35.0%–46.2%) indicated swine contact, only 12 (4.1%, CI: 1.9%–6.4%) of the veterinarians were occupied with pigs more than 50% of their working time.

**TABLE 1:** Occupational animal contact of veterinary practitioners included in the study (*n* = 293).

Animal species/group	Total *n*/% (CI)	Female *n*/% (CI)	Male *n*/% (CI)
Dogs	**264/90.1** (86.7–93.5)	**200/92.6** (89.1–96.1)	**64/83.1** (74.7–91.5)
Cats	**271/92.5** (89.5–95.5)	**205/94.9** (92.0–97.8)	**66/85.7** (77.9–93.5)
Rodents	**194/66.2** (60.8–71.6))	**154/71.3** (65.3–77.3)	**40/51.9** (40.7–63.1)
Reptiles	**72/24.6** (19.7–29.5)	**57/26.6** (20.7–32.5)	**15/19.5** (10.7–28.3)
Birds	**124/42.3** (36.6–48.0)	**94/43.5** (36.9–50.1)	**30/39.0** (28.1–49.9)
Poultry	**157/53.6** (47.9–59.3)	**113/52.3** (45.6–59.0)	**44/57.1** (46.0–68.2)
Fishes	**30/10.2** (6.7–13.7)	**20/9.3** (5.4–13.2)	**10/13.0** (5.5–20.5)
Cattle	**122/41.6** (36.0–47.2)	**72/33.3** (27.0–39.6)	**50/64.9** (54.2–75.6)
Sheep	**117/39.9** (34.3–45.5)	**73/33.8** (27.5–40.1)	**44/57.1** (46.0–68.2)
Swine	**119/40.6** (35.0–46.2)	**72/33.3** (27.0–39.6)	**47/61.0** (50.1–71.9)
Horses	**133/45.4** (39.7–51.1)	**93/43.1** (36.5–49.7)	**40/51.9** (40.7–63.1)
Wildlife	**31/10.6** (7.1–14.1)	**14/6.5** (3.2–9.8)	**17/22.1** (12.8–31.4)

Abbreviation: CI, 95% confidence interval.

The study participants indicated a duration of veterinary practice from under 1 year to more than 20 years. The majority of probands (65.5%, CI: 60.1%–70.9%) had been engaged in the veterinary profession for a minimum of 10 years. The weekly working time ranged from under 10 to more than 40 h, with 84.3% (CI: 80.1%–88.5%) working more than 20 h a week. In 25 (8.5%, CI: 5.3%–11.7%) of the households of the study participants, a person working in human medicine was living.

Hygienic habits during work, i.e., washing and disinfection of hands and/or wearing gloves, can be seen in Table 2.

**TABLE 2:** Hygienic habits of the study participants during their veterinary work (*n* = 290).

	Always *n*/% (CI)	Potentially infectious contact *n*/% (CI)	Never *n*/% (CI)
Washing of hands	**213/72.7** (67.6–77.8)	**73/24.9** (19.9–29.9)	**3/1.0** (0.0–2.1)
Disinfection of hands	**73/24.9** (19.9–29.9)	**198/67.6** (62.2–73.0)	**17/5.8** (3.1–8.5)
Wearing gloves	**53/18.1** (13.7–22.5)	**204/69.6** (64.3–74.9)	**32/10.9** (7.3–14.5)

Abbreviation: CI, 95% confidence interval.

### 3.2 Vaccination History

**FIGURE 1:**
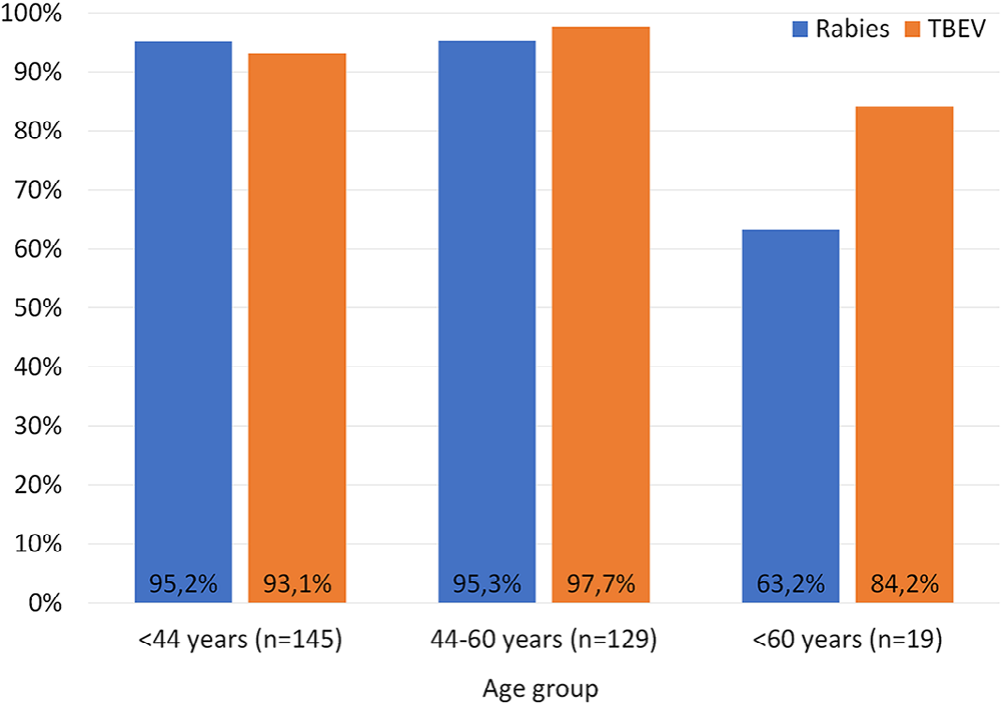
Vaccination rate against rabies and TBEV reported by the veterinary practitioners included in the study (*n* = 293), divided in age groups. *indicates significant differences to the other groups.

A vaccination against rabies was indicated by 274 (93.5%, CI: 90.7%–96.6%) of the study participants. Only a few probands had received a recent vaccination (less than 1 year ago, 5.5%, CI: 2.9%‐8.1%), most vaccinations dated five or more years back (58.5%, CI: 52.9%–64.1%). A similarly high number of the study participants (276, 94.2%, CI: 91.3%–96.7%) were vaccinated against TBEV. The vaccination dated less than 1 year back in 9.2% (CI: 5.9%–12.5%). On the other hand, 41.6% (CI: 36.0%–47.2%) were vaccinated more than 5 years ago. Significantly less study participants of age group three (60 years or older) than in the other age groups were vaccinated against rabies or TBEV (Figure 1).

### 3.3 Laboratory Findings

The rabies‐specific antibody titre among vaccinated study participants ranged from 0.0 to 122 IU/mL. A total of five individuals (1.8%) who indicated having received a vaccination had a rabies antibody titre below 0.5 IU/mL. In four cases vaccinations were allegedly more than 10 years ago, in one case 3–5 years ago. Contrarily, antibody titres of < 122 IU/mL were observed in persons who reported receiving their last rabies vaccination between 3 and over 10 years ago. No difference could be found between genders and age groups. Two out of the 19 study participants that indicated no known vaccination against rabies, had a protective rabies specific antibody titre. One was a female person aged 52 years (0.5 IU/mL), the other a male person aged 64 (2.5 IU/mL).

TBEV specific antibodies were found in 281 (95.9%, CI: 93.6%–98.2%) study participants. One sample of a vaccinated person was questionable. Of the 11 negative study participants, four were vaccinated against TBEV. On the other hand, nine TBEV antibody positive study participants stated not to be vaccinated.

A summary of the further laboratory findings can be seen in Table 3. In nine study participants, antibodies against WNV were detected, one was questionable. All except one were confirmed positive in the WNV‐competition ELISA. Antibodies against HEV were found in 33 of the study participants. The *Echinococcus* spp. ELISA detected antibodies in eight of the study participants. In the following western blot, however, the results were not confirmed. Therefore, all study participants were declared negative for antibodies against *Echinococcus* spp. No antibodies against CCHF virus or *Brucella spp*. were found in any of the study participants. A MRSA colonisation was detected in 19 study participants.

**TABLE 3:** Antibody rates and MRSA colonisation of the veterinary practitioners included in the study and the correlation with potential risk factors.

	HEV antibodies *n*/%	WNV antibodies *n*/%	MRSA colonization *n*/%
Total, *n* = 293	33/11.3	9/3.1	19/6.5
Gender			
Female, *n* = 216	21/9.7	6/2.7	11/5.1
Male, *n* = 77	12/15.6	3/3.9	8/10.4
Age groups			
< 44 years, *n* = 145	9/6.2	4/2.8	9/6.2
44–60 years, *n* = 129	19/14.7*	4/3.1	6/4.7
> 60 years, *n* = 19	5/26.3**	1/5.3	4/21.1*
Washing of hands			
Always, *n* = 213	22/10.3		11/5.2
Infectious contact, *n* = 73	11/15.1		6/8.2
Never, *n* = 3	0		2/66.7*
Disinfection of hands			
Always, *n* = 73	7/9.6		2/2.7
Infectious contact, *n* = 198	25/12.6		13/6.6*
Never, *n* = 17	1/5.9		4/23.5**
Wearing gloves			
Always, *n* = 53	7/13.2		5/9.4
Infectious contact, *n* = 204	23/11.3		12/5.9
Never, *n* = 32	3/9.4		2/6.3
Livestock exposure			
Yes	19/12.3		16/10.3*
No	14/10.1		3/2.2
Cattle exposure			
Yes, *n* = 122	15/12.3		15/12.3*
No, *n* = 171	18/10.5		4/2.3
Sheep exposure			
Yes, *n* = 117	12/10.3		13/11.1*
No, *n* = 176	21/11.9		6/3.4
Swine exposure			
Yes, *n* = 119	13/10.9		14/11.8*
No, *n* = 174	20/11.5		5/2.9
Poultry exposure			
Yes, *n* = 157	21/13.4	2/1.3	10/6.4
No, *n* = 136	12/8.8	7/5.1	9/6.6

The presence of HEV antibodies as well as the colonisation with MRSA were age‐dependent. HEV antibodies or MRSA colonisation were detected significantly less frequently in the youngest age group than in the older age groups. Most frequently, the age group above 60 years was positive. Veterinarians that indicated never to washing their hands after contact with animals were significantly more often colonised with MRSA (two out of three persons), although the number in this group was very low. Disinfection of hands after animal contact had a significant influence on the colonisation with MRSA. The more consequent the hand disinfection was executed the less colonisation was found. The use of gloves, however, did not affect the frequency of MRSA colonisation. Occupational exposure to livestock animals such as cattle, sheep or pigs had no significant influence on the occurrence of HEV antibodies. Opposite to that, MRSA colonisation was significantly more prevalent among individuals with livestock occupational exposure (Table 3). No social or occupational risk could be detected regarding the results of the WNV antibody ELISA.

## 4 Discussion

In this study, the prevalence of antibodies against selected zoonotic pathogens and the colonisation with MRSA in Austrian veterinary practitioners was determined. Although this is not a representative study, the age and gender distribution as well as the professional occupation with pets and/or livestock animals corresponds to the veterinarian statistics 2022 in Germany (Bundestierärztekammer 2023) and is probably similar in Austria.

There are no vaccination statistics against rabies available for Austria. The pre‐exposure prophylaxis is recommended in risk groups, including veterinarians. Accordingly, the high vaccination rate with 93.2% reported in this study can be explained. However, the rabies vaccination rate in veterinarians over 60 years of age was significantly lower (63.2%). Although the eradication of urban rabies in Europe is progressing (Robardet et al. 2019), the willingness for vaccination in younger persons with occupational exposure seems to increase. This is reasonable because of the possible re‐emergence of the virus as is currently seen in Poland (Smreczak et al. 2022). Only 1.8% of the vaccinated study participants had an inadequate antibody titre (below 0.5 IU/mL). In a study of Parize et al. (2021), one person (0.5%) had an inadequate immune response after a repeated booster vaccination. More than 5 years after the last vaccination, in about one quarter of the study population no protective antibody titre was detected (Parize et al. 2021). In some studies, female participants had higher antibody titres than male (Dougas et al. 2020; Parize et al. 2021). In our study, neither the gender nor the indicated vaccination time point had a significant influence on the detected rabies specific antibody titre. Two of the 19 study participants that had no rabies vaccination history had a protective antibody titre, although in the lower range (0.5, respectively 2.5 IU/mL). Since the vaccination history had been given from the memory of the persons and was not verified by checking the vaccination passport, it is probable that a rabies prophylaxis given many years ago has simply been forgotten by the persons or they did not reckon it as relevant.

The vaccination rate of the participants against TBEV was 94.2%, which is higher than in the general Austrian population (about 85%, Federal Ministry of Social Affairs, Health, Care and Consumer Protection 2024). Accordingly, a high rate of TBEV seropositive study participants were found (95.9%). There were, however, four vaccinated participants that had no detectable antibodies against TBEV. Since the vaccination data have been given only from the memory of the persons, the vaccination could have been longer ago than they remembered. In Austria, after four basic vaccinations against TBEV, boosters are recommended every 3–5 years, depending on the antibody titre. In individuals with few booster vaccinations, the probability of being detected as seronegative increases. Overall, about 13.8% seronegative individuals among vaccinated persons in Poland were found in another study (Janik et al. 2020). On the other hand, we found antibodies against TBEV in nine (3.1%) supposedly non‐vaccinated study participants. We were not able to verify in our study, if some of them had been vaccinated against TBEV although they had stated otherwise. An infection with TBEV causes clinical symptoms only in about 30% of the cases. In a study from Germany, 5.6% of the study participants had antibodies due to a former infection with TBEV (Euringer et al. 2023).

The risk for exposure to WNV in Europe increases due to climate change (Erazo et al. 2024). In nine (3.1%) of our study participants, antibodies against WNV were detected. From the data of the questionnaire, no conclusion can be made regarding the source of contact with the WNV (autochthon or imported). Similar results were found among healthy blood donors in Romania (Coroian et al. 2022). No seroprevalence data in humans are available from Germany or Austria. The WNV seroprevalence in horses is about 3.3% in Germany. Horses are supposed to be sentinels for the circulation of WNV in Germany (Gothe et al. 2023).

Swine practitioners as well as other workers with occupational swine contact are reckoned to be high risk groups for infections with HEV. In a former study, veterinarians with swine contact had developed significantly more often antibodies against HEV than other veterinarians (34% vs. 18.2%) (Taus et al. 2019). According to a meta‐analysis, the risk for infection with HEV and the seroprevalence are significantly higher in swine veterinarians than in the total population, although the overall seroprevalence varied widely between the countries and depending on the ELISA test used (Hartl et al. 2016; Mrzljak et al. 2021). In our study, we found a comparatively low seroprevalence (11.3%) among the tested veterinarians with no correlation to swine or livestock contact. There was, however, an age‐dependence with a higher seroprevalence up to 26.3% in the older age groups. Hartl et al. (2016) also detected an age‐dependence of HEV seroprevalence in the analysed European studies. Wearing gloves seemed to have an influence on HEV seropositivity in another study (Taus et al. 2019). We found, however, no effect of hygienic measures on HEV seropositivity in our study.

We found a significantly higher MRSA colonisation rate in livestock occupationally exposed veterinarians than in those not exposed to such risks (10.3% vs. 2.2%). Goerge et al. (2017) reported in veterinarians in Europe MRSA colonisation rates between 2.6% among those occupationally exposed to cattle and 45% among those occupationally exposed to pigs. Veterinarians occupationally exposed to diverse livestock had MRSA colonisation rates up to 12% which is in line with our study (Goerge et al. 2017). Although MRSA can besides its presence in cattle, sheep and pigs also be isolated from poultry (Benrabia et al. 2020), in our study no significant influence of occupational exposure to poultry or birds on MRSA colonisation of the study participants was found. This was confirmed in a study by Dahms et al. (2014) that found no MRSA colonisation in German poultry farmers. There was, on the other hand, a significantly higher colonisation rate in those with occupational exposure to pigs, although only few study participants stated to have more than 50% of their working time occupied with pigs. Sporadic contact to pigs seemed to be sufficient to colonise with MRSA more frequently (11.8% vs. 2.2% of the non‐exposed). In a former Austrian study, the occupational exposure (more than three visits in swine farms per week) of swine veterinarians led to a significant increase of MRSA colonisation (38.3% vs. 7.5% in non‐exposed veterinarians) (Taus et al. 2019). Appropriate hygienic behaviour like hand washing and disinfection led to significantly fewer cases of colonisation with MRSA, whereat wearing of gloves did not prevent the colonisation any further. In other studies, no conclusive hygienic measure could be identified that prevented occupational workers from colonisation with MRSA (Goerge et al. 2017; Taus et al. 2019).

No antibodies against *Echinococcus* spp. were found in our study, although most study participants were in regular contact with cats and dogs (more than 90%) and some (10.6%) had contact with wildlife and might therefore have been exposed. The close contact with dogs increases the risk of getting infected (Schmidberger et al. 2022). An infection risk can also occur from contacts with foxes or fox faeces (Duscher et al. 2006).

In none of the study, participants antibodies against CCHF virus and *Brucella* spp. could be detected. Accordingly, no occupational risk could be identified for Austrian veterinarians in our study. Austria is officially free from CCHF in humans (ECDC 2022) and Brucellosis in livestock (EFSA and ECDC 2024). Another study discussed the occupational risk for veterinarians of being exposed to CCHF virus in endemic regions in Turkey (Aydin et al. 2020). An occupational risk for an infection with *Brucella* spp. has been detected in a meta‐analysis which is especially relevant in countries in which Brucellosis is endemic among livestock and pets (Narimisa et al. 2024).

## Author Contributions

**Tatjana Sattler**: conceptualisation, data curation, formal analysis, investigation, writing – original draft. **Bernhard Benka**: resources, writing – review and editing. **Irene Zimpernik**: data curation, validation, writing – review and editing. **Sarah Pürrer**: data curation, validation, writing – review and editing. **Karoline Waldner**: methodology, writing – review and editing. **Georg G. Duscher**: validation, writing – review and editing. **Agnes Kuffer‐Rosewick**: resources, data curation, validation, writing – review and editing. **Jasmin Fertey**: investigation, formal analysis, writing – review and editing. **Sebastian Ulbert**: formal analysis, writing – review and editing. **Friedrich Schmoll**: project administration, conceptualisation, writing – review and editing.

## Ethics Statement

The study adheres to the criteria of the Declaration of Helsinki. The institutional review board of the city of Vienna studied the protocol and decided on 10 August 2022 under EK /22‐182‐VK‐NZ that the study did not require a formal ethical review. All participants provided written informed consent to participate in the study and to have their case details published.

## Conflicts of Interest

The authors declare no conflicts of interest.

## Peer Review

The peer review history for this article is available at https://www.webofscience.com/api/gateway/wos/peer‐review/10.1002/vms3.70485.

## Data Availability

Data are available from the authors on request.
